# Supplementary material for: Geometry of population activity in spiking networks with low-rank structure
Source: PLoS Comput Biol. 2023 Aug 7;19(8):e1011315. doi: 10.1371/journal.pcbi.1011315 (PMC10461857; doi:10.1371/journal.pcbi.1011315)
Supplement: Attachment — Submitted filename: PLOS CB reviews_responses.pdf [file pcbi.1011315.s001.pdf]

Reply to Reviewers' Comments

Reviewer #1: (original comments in blue)

In this manuscript, the authors demonstrate empirically that several interesting properties of firing rate network models with low rank structure also hold in spiking network models. This is a clear, straightforward, and useful study that fills a gap in the literature. Pending some minor edits suggested below, I feel that this manuscript is appropriate for publication in PCB.

We thank the reviewer for their supportive and constructive comments. We have implemented all the corrections as detailed below.

1) In the first couple of pages of Results, the models are described qualitatively in words, but equations for the models are not given. This is sometimes a reasonable choice of style for papers intended for an audience of biologists when the details of the model are secondary to the biological interpretation of the results. However, the audience for this manuscript is computational neuroscientists and the details of the model are central to the paper. Readers who are not familiar with this class of low-rank network models will need to flip back-and-forth between the Results and Methods to understand what is really going on. Whereas, most computational neuroscientists would only need a quick glance at a few equations up front in the Results section to understand the basic construction of the model.

Furthermore, even in the Methods, some details seem to be missing or at least they are difficult to infer from what is written.

I suggest that the authors put more details of the model in the Results section, particular in the first 2-3 pages where the model(s) are introduced. Some specific suggestions follow:

1a) Second sentence of the Results ("We quantify ..."):
Replace this sentence with Eq 12 from Methods (or the equivalent equation in vector form) and also add the equation r_i=phi(x_i) (OR r=phi(x) if using vector form). You can still explain what each variable represents, but it's much clearer to have the actual equations up front.

We have now copied the equation for the rate model to the beginning of the results (new Eq. 1).

I personally prefer the vector form of the equations (instead of the component form used currently), but the authors can choose whichever form they prefer.

We understand this is a matter of personal preference. For consistency with previous manuscripts from the lab, we would rather keep the component form.

1b) In Methods, "The recurrent connectivity matrix J consists of a rank R structure P":

It is not clear what is meant by "consists of" here. Is J=P? If so, then why have two names for it? Or is J sparse with P the connection probability matrix? Or J=P+noise with J dense? Write out exactly how J is related to P. If J=P, then maybe it's better to have just one name for it?

Put this more precise definition of J and its relationship to P in the Results (replacing or

complementing the discussion surrounding Eq 1, which also does not clarify the relationship between J and P).

We thank the reviewer for pointing out this confusion. For the rate model, the connectivity is P, while for the spiking model the connectivity is J=J^{EI}+P.

We have now clarified this point both in the Results and the Methods, and changed the name of the connectivity in the rate model to P (new Eq. 1).

1c) Are the J matrices generated in exactly the same way for the rate and spiking models? For spiking models, J=JEI+P where JEI is a sparse EI network (Eq 16). If J is not generated in exactly the same way for the rate models, then it is better to give the two J's different names and make their definitions and relationship more explicit and precise in the Results section.

I do not think that the equations defining the spiking model dynamics need to be given in Results. Those are fine just in the Methods, but the connectivity matrices should be defined.

As mentionned above, for the rate model, the connectivity is P, while for the spiking model the connectivity is J=J^{EI}+P. We have now clarified this point in both Results and Methods, and modified the nomenclature as suggested by the reviewer.

2) Line 120 "Statistically identical low-rank connectivity structure"

Related to the last comment above, if J is generated in the same exact way then I think this is misleading. I see that "identical low-rank structure" could be interpreted to mean that only the low-rank part if statistically identical, but the phrase could also be interpreted to mean that the matrices themselves (which have a low-rank structure) are statistically identical. If the two J matrices are not statistically identical, please rephrase. For example, "Connectivity structures with statistically identical low-rank components" or something similar.

Only the low-rank parts are statistically identical. We have followed the reviewer's suggestion to clarify this point (l.104).

3) Line 229: "independently of the activity regime"

This is a little bit stronger than what you have shown. There are inevitably activity regimes that you have not tested. Instead, it would be better to write something like "in both the AI and SI regimes"

Indeed this was too strong. We have now toned it down to "hold in different activity regimes" (l.226-227).

4) Lines 245-247:

Eq 4 here looks like an explicit equation for \kappa, but it is an implicit equation. While the equation is correct, it might mislead some readers who interpret it as an explicit equation. Instead of writing "\kappa is therefore given by" you can write "\kappa therefore satisfies". And,

after Eq 4, you can write "where x_i is the steady state value of x_i(t)" or something similar.

Modified as suggested.

5) Lines 425-428 ("We expect that our findings hold..."):

I agree with the authors' expectation expressed here, but only under the additional assumption that the dynamics being studied should be related to the "discrete outliers" and not to the details of the eigenvalues inside the bulk itself. One can easily design examples where the low rank part does not capture the dynamics of interest. If the authors agree with this caveat, it might be worth mentioning. But I won't quibble if the authors disagree and choose not to incorporate this suggestion.

This is an important point, we have rephrased this sentence to say that the dynamics should “rely on discrete outliers” (lines 420-422):

We expect that our findings hold as long as dynamics rely on discrete outliers in the the eigenspectrum (or singular value distribution), in which case the connectivity can be accurately approximated by a low-rank structure.

Reviewer #2: (original comments in blue)

Chimes et al. present a comparison of the dynamics of randomly connected rate networks with low-rank connectivity components to those of excitatory-inhibitory integrate-and-fire networks with corresponding low-rank connectivity components. They find similar bifurcations in the spiking network as in the rate network. My major comments relate to discussing the theoretical basis for the analogy between the rate and spiking networks, how the regime of the underlying spiking network impacts the low-rank dynamics, and whether this impacts the variability of activity in the two networks.

We thank the reviewer for their constructive suggestions that we address in detail below.

1) The authors' hypothesis that a rate network can be viewed as a high-dimensional mean field theory for a spiking network has some support in the derivation of rate networks as mean-field descriptions (i.e., neglecting fluctuations) of switching networks (e.g., Ginzburg & Sompolinsky 1994; Buice et al., 2006), and more recently in integrate-and-fire networks with stochastic spike emission (Ocker 2022).

A systematic mean field approximation of a deterministic integrate-and-fire network by a rate network is unknown (at least to me). The closest is the classic diffusion approximation. The spectral approach to the Fokker-Plnack dynamics in the diffusion approximation (Mattia & del Giudice 2006) might provide a more principled choice of rate network to match to the integrate-and-fire network. The sparse connectivity, weak connection strength (J=0.1 mV or 0.2 mV), and injected independent white noise suggest that the Fokker-Planck approach should be applicable. (While the Fokker-Planck equation resulting from the diffusion approximation is usually viewed as describing the distribution of membrane potentials across a population, it has also been used to describe the statistical density of single-neuron membrane potentials-for example in Trousdale et al., 2012.) In the absence of such a justification, why compare the rate network to an integrate-and-fire network instead of a compartmental network or some other model?

We thank the reviewer for pointing out mathematical relationships between spiking and rate models. We fully agree that a systematic mean-field approximation of integrate-and-fire networks by rate networks is unknown. We now state this explicitly in the introduction (line 26: "a systematic mathematical reduction of arbitrary spiking networks to rate models has been elusive"). We have also added all the references suggested by the reviewer, and a couple of additional ones that were missing in the previous version (eg Montbrio, Paz & Roxin 2015).

In absence of a rigorous mathematical derivation, our comparison between spiking and rate networks is motivated by heuristics used by a number of previous works cited in the Introduction:[Tetzlaff et al. 2012;Ostojic, 2014;Harish and Hansel 2015;Nicola and Clopath,

2017;Kim and Chow, 2018;Kim et al., 2019]. We now clarify that this is a heuristic, and not a rigorous mathematical derivation (lines 24-26).

On a related note, the use of slow filters to map from the activity of a spiking network to a rate network also has some earlier precedent in the work of Pinto et al., 1996, who derived rate networks of this form under the assumption that synaptic interactions are slow relative to the membrane timescale.

We have added the reference to Pinto et al, but please note that in our case the synapses are instantaneous as in Brunel 2000 (Eq. 16). The slow filter is only used to compute single-neuron firing rates from spike trains, not for interactions between neurons.

2) Since the spiking and rate networks are quite different systems on their face, the existence of shared bifurcations is surprising. The classic heuristic derivations of rate models apply most closely to asynchronous irregular networks; do the dynamics of figures 5-7 depend on the regime of the spiking network?

We thank the reviewer for this question. We performed additional simulations, which show analogous bifurcations outside of the asynchronous irregular regimes. These results have been added in a new Fig 8.

We also added the following text (l.290-292):
As for transient dynamics, these findings held also outside of the asynchronous irregular regime, when the neurons were sparsely synchronized (Fig. 8 A) or fired regularly (Fig. 8 B).

and (l.362-364):

These findings did not rely on the network being in the asynchronous irregular regime, as we observed similar dynamics when the neurons were sparsely synchronized (Fig. 8 C,E) or fired regularly (Fig. 8 D,F).

3) Beyond showing that the LIF network has similar bifurcations of equilibria as the rate network, it would be useful to examine how their statistics differ. Do second-order statistics in the two networks (single-neuron and joint variability) exhibit the same trends in the two networks? And, do these depend on the underlying regime of the spiking network?

In this work we focus on deterministic rate networks that are meant to represent average activity. Second-order statistics, and in particular cross-correlations, could be compared by adding noise in the rate network [Tetzlaff et al. 2012], but this lies beyond the scope of the present study.

Minor comments:

The mean excitation and inhibition could be viewed as a low-rank component of the spiking network's connectivity. Does the full connectivity matrix, $J^{EI} + P$ in Eq. 16, still obey Dale's law?

In the original submission, the full matrix in the spiking network did not obey Dale's law, and was not sparse because of the low-rank term, which is all-to-all. Note however that the non-zero terms in the random part of the connectivity J^{EI} are much stronger than the entries of the low-rank component P (variance $\sigma_n*\sigma_m/N$).

Setting to zero entries P_{ij} for which $J^{EI}_{ij} = 0$ is therefore sufficient to enforce Dale's law. The resulting, sparsified matrix P_{ij} is not low-rank anymore, but the predictions of low-rank theory still hold in this case in rate networks up to high levels of sparsity (Herbert and Ostojic 2022).
We have now added a figure (new Fig 4) showing that enforcing Dale's law and sparsity does not modify the results in spiking networks. We also added the following text (l.157-165):

Because the low-rank structure $\bm{P}$ is generated using Gaussian connectivity vectors, the full connectivity matrix obtained by superposing it with the random component $\bm{J}^{EI}$ does not obey Dale's law and is not sparse. The entries of $\bm{P}$ are however much weaker (standard deviation $\sigma_n*\sigma_m/N$) than the non-zero elements of $\bm{J}^{EI}$. Setting to zero entries of $\bm{P}$ for which $\bm{J}^{EI}$ is zero was therefore sufficient to enforce Dale's law in the full connectivity matrix. The resulting sparsified matrix $\bm{P}$ is not low-rank anymore, but for rate networks the predictions of low-rank theory still hold up to high values of sparsity \cite{herbert2022}. We verified that the results in the spiking network were unchanged by including sparsity in the low-rank term and Dale's law in the full connectivity (Fig. 4).

Line 140: What is the linear regime of the network?
We have rephrased this to "if the response was weak and therefore approximately linear" (l.124).

Line 134: "i.e." is usually followed by a comma (as is "e.g.").

corrected

Line 270: Bistability between low and high states was also a feature of the early model of Ashby et al. 1962, as well as Amari (1971),

and a classic feature of the wilson-cowan equations (wilson & Cowan 1972).

We have now added these references in the relevant part of the Results.

Slow filters let rate network describe spiking network - similar to earlier works with slow synapses giving a rate description (e.g., harish & Hansel, ermentrout)

Please note that in our case the synapses are instantaneous as in Brunel 2000 (Eq. 16). The slow filter is only used to compute single-neuron firing rates from spike trains, not for interactions between neurons. We nevertheless cite the mentioned references.

LIF networks with only low rank structure (?) scaling? All to all?

We are unsure what the question is here, but the low-rank connectivity structure is all-to-all with scaling indicated in Eq.1. As noted above, the results still hold if sparsity is introduced in the low-rank part (new Figure 4).

There were some references to “spiking noise” but the underlying spiking model is deterministic.

We have replaced “spiking noise” by “variability in spike times”. Note that the LIF neurons receive independent fluctuating inputs as in Brunel 2000, and the initial condition in the membrane potential contributes additional variability.

Reviewer #3: (original comments in blue)

The authors add a low-rank matrix to the EI matrix of a spiking neural network and show that filtered spike trains follow similar low-dimensional dynamics as rate neurons connected by the same low-rank matrix. Various examples comparing rates networks with spiking networks are provided.

I have some concerns:
A) I would expect to see a comparison of the variance of the synaptic inputs into a neuron due to the EI matrix and that due to the low-rank matrix. What happens to the results if one increases or decreases the variance of the n component (sigma_n in Table 4). I suspect that currently the low-rank component dominates over the EI part of the weight matrix. The authors should titrate the effect of each component. Currently only removing the EI component has been studied in Fig 4G-I, but this is moot if the effect of the EI connectivity is already minor. Indeed, it appears that the hypothesis that neurons with Poisson / AI spiking are needed to represent rate neurons is not borne out, since removing the EI matrix yields similar low dimensional dynamics.

We thank the reviewer for bringing up this aspect that we had not sufficiently emphasized in the original submission. The low-rank part is given by Eq.1. For unit-rank networks, the standard deviation of elements of P is therefore sigma_n*sigma_m/N, while the elements of J^{EI} do not have a 1/N scaling (as in Brunel 2000). Since N=12500 in spiking networks, the elements of P are orders of magnitude smaller (in absolute value) than the non-zero elements of J^{EI}. In most figures, the E-I part therefore largely dominates, so that removing this part (Fig 4G-I) is in fact the relevant comparison.

We now added the following note on lines 142-144:
Note that, because of the $1/N$ scaling in Eq.~\eqref{Eq_Jij}, the magnitude of elements of $\bm{P}$ was much smaller than the non-zero elements of $\bm{J}^{EI}$ that were independent of N \cite{Brunel2000}. The excitatory-inhibitory part of the connectivity therefore controlled the firing regime of the network.

and on lines 216-219:
As noted above, on the level of individual synapses this removed the dominant part of the connectivity, as the magnitude of non-zero terms in J^{EI} was much larger than the magnitude of terms in P.

As the reviewer points out, our original hypothesis was that the E-I part and the AI regime are needed for the predictions of rate networks to hold. To our surprise, we instead found that the predictions hold even in absence of the E-I part, ie in a larger dynamics range than expected. This implies that the original hypothesis was too restrictive, rather than incorrect. We now point this out more explicitly in the Discussion (l.428-429):

Our original hypothesis that asynchronous irregular activity is required appears to have been too restrictive.

B) Even though the authors start with an EI matrix that satisfies Dale's law, it seems the final weight matrix would violate Dale's law since J=0.1mV and sigma_n=20mV. Can the authors maintain Dale's law and yet obtain their results? This will possibly require using a much smaller sigma_n and clipping weights. In either case, further study and discussion on this is needed for relevance to biology, especially since the authors start with an EI matrix.

The reviewer is absolutely correct. In the original submission, the full matrix in the spiking network did not obey Dale's law, and was not sparse because of the low-rank term. However, since the non-zero terms in the random part of the connectivity J^{EI} are much stronger than the entries of the low-rank component P, setting to zero entries P_{ij} for which J^{EI}_{ij} = 0 is in fact sufficient to enforce Dale's law, and no clipping is needed. The resulting sparsified matrix P is not low-rank anymore, but previous works has shown that the predictions of low-rank theory still hold in this case in rate networks (Herbert and Ostojic 2022).
We have now added a figure (new Fig 4) showing that enforcing Dale's law and sparsity does not modify the results in spiking networks. We also added the following text in the Results (l.157):

Because the low-rank structure $\bm{P}$ is generated using Gaussian connectivity vectors, the full connectivity matrix obtained by superposing it with the random component $\bm{J}^{EI}$ does not obey Dale's law and is not sparse. The entries of $\bm{P}$ are however much weaker (standard deviation $\sigma_n*\sigma_m/N$) than the non-zero elements of $\bm{J}^{EI}$. Setting to zero entries of $\bm{P}$ for which $\bm{J}^{EI}$ is zero was therefore sufficient to enforce Dale's law in the full connectivity matrix. The resulting sparsified matrix $\bm{P}$ is not low-rank anymore, but for rate networks the predictions of low-rank theory still hold up to high values of sparsity \cite{herbert2022}. We verified that the results in the spiking network were unchanged by including sparsity in the low-rank term and Dale's law in the full connectivity (Fig. 4).

Have the authors made all data and (if applicable) computational code underlying the findings in their manuscript fully available? (original comments in blue)

Reviewer #1: **No:** A github link is included, but the repository is empty.

Reviewer #2: None

Reviewer #3: None

We will make the code available upon acceptance.
